# Disturbance of Intentionality: A Phenomenological Study of Body-Affecting First-Rank Symptoms in Schizophrenia

**DOI:** 10.1371/journal.pone.0073662

**Published:** 2013-09-03

**Authors:** Dusan Hirjak, Thiemo Breyer, Philipp Arthur Thomann, Thomas Fuchs

**Affiliations:** 1 Section Phenomenological Psychopathology and Psychotherapy, Department of General Psychiatry, Center of Psychosocial Medicine, University of Heidelberg, Heidelberg, Germany; 2 Structural Neuroimaging Group, Department of General Psychiatry, Center of Psychosocial Medicine, University of Heidelberg, Heidelberg, Germany; Baylor College of Medicine, United States of America

## Abstract

**Objectives:**

In 1950, Kurt Schneider proposed that a considerable number of schizophrenia patients develop first-rank symptoms (FRS). In such cases, patients report made experiences, replaced control of will, thought insertion, broadcast or withdrawal and delusional perception, respectively. Although a number of recent studies tend to explain FRS in terms of neurobiological and neuropsychological processes, the origin of these symptoms still remains unknown. In this paper, we explore the subjective experience of patients with the following two FRS: (1) "made" impulses and (2) “made" volitional acts.

**Method:**

The method applied for the study of two FRS consists first in the overview of psychiatric and philosophical literature and second in the further investigation of subjective experience in patients with FRS. Psychopathological and phenomenological aspects of FRS are discussed by means of patient cases.

**Results:**

We discovered a profound transformation of intentionality and agency in schizophrenia patients with body-affecting FRS. This concept offers an insight into the interrelatedness between particular FRS.

**Conclusion:**

We propose that the subjective experience of schizophrenia patients with body-affecting FRS is rooted in the disturbance of intentionality and diminished sense of agency. This theoretical account of body-affecting FRS will open up new directions in both phenomenological and neurobiological psychiatric research.

## Introduction

### 1: Clinical background

Schneiderian first-rank symptoms of schizophrenia (FRS) designate 11 extraordinary phenomena not occurring in “healthy” human self-experience. Hence they illustrate a profound change in self-experience that may be attributed to a disturbed sense of mineness [1,2]. Patients with schizophrenia experience these phenomena as not belonging to themselves, overwhelming and made from outside. As Schneider pointed out, this group of symptoms may also be regarded as “a loss of the very contours of the self” [2]. According to Mellor, 72% of patients suffering from schizophrenia experience at least one first-rank symptom [1]. In the modern criteriological diagnostic systems such as DSM-IV and ICD-10, FRS play a central role, being part of the specific diagnostic concept of schizophrenia. Throughout the 20^th^ century, psychiatric research has attempted to explain FRS and schizophrenia by using different scientific methods [3–16]. In the last three decades, a wealth of neurobiological and neuropsychological studies has supported the assumption that the exploration of FRS may help to understand both the development and the psychopathology of schizophrenia [17–26]. Despite these encouraging scientific results, we are still lacking knowledge about the origin of FRS, and it is an ongoing matter of debate whether these symptoms are pathognomonic of schizophrenia psychopathology and thus relevant for the clinical diagnosis of schizophrenia [27–29].

In the last two decades, in order to increase the precision and reliability in diagnosing mental illness, psychiatry has experienced a paradigm shift: It moved away from subject-oriented explanations of mental disorders grounded on the comprehension of the patients’ experiential world towards new nosological constructs on rigid descriptions and operationalized, but inflexible criteria. This seems to be one of the reasons why today’s studies are often lacking homogenous patient groups due to the oversimplified definition of schizophrenia and its related symptoms [30,31]. Therefore, psychiatry as a scientific domain needs to go beyond stiff nosology, try to capture what patients really experience and delineate a precisely defined phenotype in order to identify endophenotypes underpinning schizophrenia spectrum disorders [14,32–38].

### 2: Historical background

In the history of psychiatry, a number of psychiatrists were interested in the philosophical explanation of anomalies of self-experience. Their descriptions were not restricted to what is reported verbally by the patients. Rather, they also tried to analyze the altered basic structures of experience themselves, such as temporality, spatiality or intersubjectivity. However, these authors did not yet make use of the term “intentionality”, but employed terms with similar meaning. In the late 19^th^ century, French psychologist Pierre Janet introduced a disordered mental state which he called “abaissement du niveau mental” [39,40]. In 1904, Austrian psychiatrist Erwin Stransky employed the term “intrapsychic ataxia” (incongruity between affect and cognition) to describe certain symptoms of “dementia praecox” [40,41]. Somewhat later, on the basis of various case studies of disorders of concentration, rational thinking and volition, Berze elaborated a syndrome of diminished awareness or mental activity which he called “*hypophrenia*” [42,43].

After the introduction of phenomenology at the beginning of the 20^th^ century, many psychopathologists analyzed essential phenomena of schizophrenia by means of Husserlian concepts [36,40,42,44] and employed the term *intentionality*. Beringer, a German psychiatrist, was the first to use this term in order to explain formal thought disorders and impaired language in schizophrenia patients. He assumed that these patients suffer from a reduction of the “intentional span” [45,46]. In parallel, Gruhle described formal thought disorders and delusions as being rooted in a “lack of teleological discipline”, implying the term “intentionality” [42]. Later on, Blankenburg studied the content and structure of experience in patients with hebephrenic schizophrenia by means of Husserl’s phenomenology [47]. According to his theory, patients with schizophrenia suffer from a “disturbance of the transcendental organization” and experience a disorder of the framework of *common sense* [40]. A further contribution to understanding schizophrenic self-experience was made by Mundt, who postulated that the term intentionality means “the ability to build up and maintain a coherent subjective world of patterns of meanings and purposes” [40, p. 39, 48, 49]. According to his psychopathological concept, both positive and negative symptoms in schizophrenia may be derived from a break-down of the patient’s intentionality. In this view, the development of systematic delusions means a re-establishment of meaning after the collapse of intentional performances [40,48]. More recently, Wiggins understood the initial stages of schizophrenia as being rooted in the weakness of the intentional synthesis [44]. Sass and Parnas assume schizophrenic self-experience to be derived from a profound transformation basic self-experience (and correspondingly a disturbance of the “intentional arc” [50]) which leads to diminished self-affection and hyperreflexivity [12,51]. Taken together, a phenomenologically grounded approach to schizophrenia seems a valid instrument to investigate these basic dimensions of subjective experience.

Different theoretical approaches mentioned above have been used to explain FRS in schizophrenia and there are many historical and modern models of these symptoms [8,18,52–54]. However, none of the authors has applied Husserl’s or Merleau-Ponty’s concept of intentionality explicitly to the experience of FRS. In this article, we use the phenomenological method [16,38,55] to investigate the subjective experience of patients with two body-affecting FRS. For this purpose, the theory of intentionality and Gallagher’s concept of the “sense of agency” (SA) [56] will be adopted to provide a descriptive account and theoretical framework for the investigation of the following two FRS:

1. *“Made” impulses (drive)*. In this case, patients experience a powerful impulse to carry out an action which they feel stems from an external agent [1].
2. *“Made” volitional acts*. These include phenomena such as made actions and replaced control of one’s will. Such actions are executed by the patients’ bodies, but experienced as actions of an external agent [57–60].

### 3: Intentionality and the sense of agency

From a phenomenological point of view, consciousness is not self-enclosed, but open to objects in the world, it has a world-involving character, i.e. it is intentional [61]. For Husserl, intentionality is the essential and intrinsic aspect of consciousness [62], “like a universal medium which bears in itself all mental processes, even those which are not themselves characterized as intentive” [63]. Our mental acts are always directed to something (“directedness”) and they are about something (“aboutness”) [64]. However, we are not simply aware of an object, rather we experience it within the horizon of a universal “as-structure”. We always hope, smell, see, desire, remember or fear something *as something*, i.e. in a pre-structured and specific way; it is necessarily an experience of a specific type [63,64]: watching a movie, celebrating a birthday, loving a friend, smelling a rose, paying a bill, saying a word etc. [64]. Therefore, each conscious act constitutes its specific object in a certain mode of givenness. In Husserl’s terminology, each mental process has a “material”, which is given in a specific “quality”, e.g. a cake that appears as attractive and desired. This a priori correlation constitutes our openness and relationship to the world [50]. Furthermore, intentionality implies the meaningful interaction between an embodied subject and objects in the environment. Merleau-Ponty speaks of the “intentional arc” [50] which is a mobile vector issuing from the body in all directed actions, providing an orientation towards any object in the world that we are engaged with [50,51,65].

Husserl [66] further describes the passive synthesis of *gestalt* formation based on the fact that all directed bodily experiences and movements are constituted as “consciously performed intentional acts“ [36, p. 82, 44]. These are dynamic self-organizing processes connected within the framework of activity and founded in a passive synthesis: “…all activity essentially presupposes a foundation of passivity as well as an objectlike formation that is already pre-constituted in it.” [66, p. 276]. As Husserl further writes: “the whole of conscious life is unified synthetically” [67, pp. 80-81]. Consequently, the passive synthesis of *gestalt* formation in bodily acts is a dynamic constitution of multiple aspects of mind and body unified in a meaningful way. For example, if I want to pick up a cup, the decision and intentional effort to do so is conjoined with the kinaesthetic experience of moving my arm and hand, and with the visual perception of cup, arm and hand. At the same time, the perception, decision and intentional effort guarantee the directed bodily movement to be experienced as a coherent *gestalt* [44].

Furthermore, in his writings from the *Ideas I* onwards, Husserl [63] developed an account of intentionality in which two moments of the a priori correlation are distinguished: the *noesis* or *noetic act* (sinnbildender Bewusstseinsakt) and *noema* or *noematic sense* (Sinngehalt des Bewusstseinsaktes). Every act consists of noetic moments, which can be described as “directions of the regard of the pure Ego to the objects ‘meant’ by it, owing to sense-bestowal to the object which is ‘inherent in the sense’ for the Ego” [63, p. 214]. This means that every mental process includes “in itself something such as a ‘sense’” [63, p. 213]. Noesis always incorporates noema: “Perception, for example, has its noema, most basically its perceptual sense, i.e., the perceived as perceived”, “the remembered as remembered” etc. [63, p. 214]. Both poles of intentionality are *a priori* conditions of all *object-full* experiences and their correlation characterizes consciousness [84]. Moreover, they constitute our relationship to the world and guarantee the tacit world-embeddedness of the subject [36].

Finally, intentionality is double-layered. The basic level can be called “operative or bodily intentionality” [51, p. 136, 65]; it is the pre-reflective and procedural experience which has as a main phenomenal component the character of *ipseity*. *Ipseity* or basic self-awareness (“the self-feeling of one’s self” [57, p. 122]) is a medium in which any experience, mental state or intentional action is embedded [10,68]. Ipseity mediates the first-personal mode of perception and experience. It enables the subject “to be affected by an object (hetero-affection)” and to subsequently take action towards it [51]. The second type of intentionality is founded on this stratum of consciousness and is often called “*active* or *explicit intentionality*” [51, p. 136]. It enables consciously directed actions [51]. The explicitness of this mode of consciousness entails the possibility to make one’s own mental processes thematic in a volitional manner, i.e. to reflect on one’s own experiences and their contents.

Based on the medium of ipseity, I experience my own thinking, feeling, perceiving, moving etc. immediately, noninferentially from the first-person perspective or as mine. According to Gallagher [56], the notion of mineness can be split into a sense of ownership (SO) and a sense of agency (SA). SO is the feeling that the body as a whole or functional units within the body, such as a limb one is moving, belongs to oneself. The subject simultaneously *is* the body (*Leib*), but also *has* the body (*Körper*). This double-aspectivity of the body, with the SO being more a feature of the second aspect, has been a central motive in phenomenological anthropology [69]. SA, on the other hand, is the feeling that the subject is the agent who is generating and performing his intentional actions [56]. Gallagher goes even further and distinguishes between an experiential, pre-reflective SA and an attribution of agency. He argues that the higher-order SA depends on the first-order experience of agency. For example, if a subject tries to pick up a cup, he must first have a sense of moving his arm. This first-order phenomenal experience is implicit, embodied and non-conceptual. The higher-order SA is mirrored in the fact that the subject is able to control and attribute the agency to himself [64].

### 4: Aims of the study

The overall aim of this study is to define the fundamental phenomenological pattern of the two body-affecting FRS. Through the lived body, the subject tacitly participates in the field of experience, interacts within the world [70–72] and also experiences himself as a bounded, temporally persistent entity [73]. Recent psychiatric research on individuals in the prodromal phase of schizophrenia has shown that fundamental disturbances of the lived body (concerning the feeling of mineness) may precede the development of more superficial positive symptoms such as FRS [74]. Therefore, our phenomenological explanation of the two FRS accounts for the relationship between disorders of pre-reflective self-experience and symptoms of acute schizophrenia [75]. Moreover, we describe the transition from non-psychotic anomalies of bodily experience to full-blown disorders of agency such as delusions of alien control. In this article, we put forward the hypothesis that the aforementioned two FRS can be seen as expressions of a disturbance of intentionality and agency [76, pp. 132-153, 273-279]. We also hypothesize that the two FRS are, although clinically different, interrelated and of a very similar phenomenological structure being rooted in the disorder of basic self-awareness. Referring to research by Klosterkötter [52,54,77] and Köhler [78], we describe their emergence as a sequence of four stages, leading from abnormal bodily sensations to delusions of being controlled. Since anomalous bodily experiences are considered to be potential markers of beginning schizophrenia [79,80], our study will help to better understand the experiential core *gestalt* of the prodromal phase of schizophrenia.

## Methods

In this paper, we will present descriptions of schizophrenia patients with body-affecting FRS. Four patients described [58,75] in this paper were treated in the Department of General Psychiatry, University of Heidelberg. They met the DSM-IV-TR criteria for either schizophrenia or schizoaffective disorder. All patients underwent a comprehensive psychiatric evaluation. Two patients [75] took part in a larger project (DISCOS, Disorders and Coherence of the Embodied Self) and underwent a semi-structured qualitative interview developed by Parnas and colleagues [68]. The EASE interview (Examination of Anomalous Self-Experience) focuses on a phenomenological assessment of altered self-experience with respect to five domains: (1) cognition and stream of consciousness, (2) self-awareness and sense of presence, (3) bodily experiences, (4) demarcation, and (5) existential orientation. The main focus in our study lies on the bodily experience of these patients. In addition, all patients were thoroughly explored by two experienced psychiatrists (D.H. and T.F.) in order to assess their subjective experiences before full-blown psychosis. In our study, we were mainly interested in the 1^st^-person perspective and the form and structure of conscious experience. Moreover, our aim was to grasp the experiential framework of the two body-affecting FRS and to extract the four stages of their development. We used word-by-word descriptions to understand the perspective and experience of patients with FRS. In order to protect the patients’ anonymity, relevant personal information was changed.

Besides the four patient vignettes from our own studies, we also searched for case reports describing schizophrenia patients with body-affecting FRS in the historical and modern psychiatric literature. We identified several descriptions illustrating fundamental disturbances of the self (concerning the feeling of mineness and the sense of agency) and body-affecting FRS in schizophrenia. For the purpose of phenomenological analysis, we cite several patients’ statements from modern and historical psychiatric literature.

In general, the phenomenological approach focuses on the investigation of the structure of patients’ subjective experience and the core *gestalt* of schizophrenia spectrum disorders [81, p. 3]. Therefore, both FRS will be presented in relation to the patient, his lived body, various modes of consciousness and engagement with the world. The structure of the FRS will be formalized and arranged into basic phenomenological categories such as basic and higher-level self-experience, embodiment, temporality, spatiality, intersubjectivity and, most of all, intentionality and sense of agency (SA). We employ Husserl’s and Merleau-Ponty’s concept of intentionality, regarding it as the basic characteristic of conscious experience and as the presupposition for an embodied subject to be able to understand, perceive and experience the environment in which it lives and acts. Suggesting an intrinsic link to motor intentionality, we also make use of Gallagher’s theory of SA [64]. In order to grasp the patient’s anomalous experience and the fundamental patterns underlying the two FRS, we pose the question: What it is like to experience an emergence of “made” impulses and alien volitional acts?

## Results

### A phenomenological approach to body-affecting first-rank symptoms

In the sections above, all elements have been described that are needed to analyse the development of body-affecting FRS. There are four stages that should be distinguished in principle (see also [82]):

A Weakening of operative intentionality
B Hyperreflexivity and objectification of bodily actions
C Dissociation of operative and active-explicit intentionality
D Alienation and externalisation of agency

The first three levels illustrate the phenomenological characteristics that occur in the pre-psychotic phase of schizophrenia and in schizotypal disorders. The last stage describes the transition to full-blown psychosis.

#### A: Weakening of operative intentionality

In a healthy mental state, the subject is implicitly aware of the body’s movement and the source of directed actions. It is self-present and acts in the first person perspective. Such tacit experience is an essential presupposition for the subject to interact with his environment. Now the prodromal phase of schizophrenia involves subtle, but often progressive alterations of such basic bodily experience, namely a profound disturbance of pre-reflective self-awareness or ipseity. From a phenomenological point of view, subjects in this state experience an existential insecurity and a diminished feeling of being a demarcated self:

(1) “I just don’t feel that I belong (…) as if I am different in some way (…) I just feel that I feel too much compared to other people (…) as if *I* *belonged* in another time or another place or something.” [74, p. 365]
(2) “I constantly have to ask myself “who am I really?” It is hard to explain … most of the time, I have this very strange thing: I watch myself closely, like, how am I doing now and where are the “parts” – in quotation marks. And that occupies me so much, to think about my condition, because it is not just one condition, it is always more conditions, that is exactly what is not functioning.” [75, p. 329]

The first patient is afraid of losing himself and becoming substantially different from the others. The second patient describes a fragmentation of self-presence: On the one hand, he experiences a feeling of not being an embodied subject anymore; on the other, he observes his feelings more carefully than he did before. Despite the insufficiency of the pre-reflective experience of one’s own body [51], subjects at this stage experience their bodily movements as still belonging to them (sense of ownership). On the other hand they are missing an adequate control over these actions (sense of agency). The patients are no longer auto-affected to the same degree and therefore not able to perform their intentional bodily acts properly:

(3) “I thought a lot about everything, my every movement … like things you usually take for granted, like now I take the toothbrush, now I ... it is just something you do, it is not something you tell your body to do, it is just something the brain automatically does, right, it is as if that function has been blurred so that I kind of had to tell myself to do some of those things or just had to think hard about it, or something, I don’t know, there were many times where I-I think that many of the things I took for granted just became much more difficult, even the simplest thing became incredibly difficult.” [74, p. 366]
(4) “I find it very difficult to do things now, just everyday things like shaving, things you do immediately you get up. Just things I used to do without thinking, like hanging your coat up or taking your tea. I am very easily put off now – by noises or people speaking to me. It’s trying to concentrate on two things. Sometimes I have just to cut everything short and sit down.” [83, p. 239]

From a phenomenological point of view, in both cases (3 and 4) the movements are not embedded in basic self-experience anymore. The patients are slowed down and experience everyday actions as disintegrated and unreal. They have to intensively concentrate on every single movement, because otherwise they would not be able to fulfill their intentions. Thus, they illustrate the weakening of operative intentionality of movement, or the disturbance of the tacit dimension of experience. However, patients at this stage are still able to compensate for the disturbances through intensive self-monitoring. This phenomenon will be described in the next paragraph.

#### B: Hyperreflexivity and objectification of bodily acts

The weakening of operative intentionality leads to an increasing gap between the two layers of intentional consciousness described above and to an increased self-monitoring. There is a correlation between the *decrease* on the first-level pre-reflective self-awareness and the compensatory *increase* of activity on the second-level reflective self-consciousness. If bodily movements are not embedded in basic self-presence, patients begin to ponder intensively about each single action. Four prodromal patients who later developed schizophrenia reported disturbances of their motor functions:

1. “None of my movements come automatically to me now. I’ve been thinking too much about them, even walking properly, talking properly and smoking – doing anything. Before they would be able to come automatically.” [83, p. 239]
2. “If I do something like going for a drink of water, I’ve to go over each detail – find cup, walk over, turn tap, fill cup, turn tap off, drink it.” [83, p. 239]
3. “(...)it was one of those things I did when I was feeling bad ... I had the tendency to study, well, this thing about from the thought and from the brain to send the movement to the arm, I could think – think all the way, it was as if I could not make a movement without having the brain with me, it was not something – I could not do things you take for granted(...)” [74, p. 366]
4. “There were periods in which I felt extremely badly coordinated, when I just made a movement with the arm and the arm had moved further than I wanted it to move. But I also found myself to be extremely clumsy, somehow, when walking. I therefore constantly observed my walking and my movements … Climbing the stairs was also very extreme, when you need a bit of concentration and a feeling of balance. I really thought each step after the other, as it were, each movement…” [75, p. 330]

In these cases we face a weakness of operative intentionality on the one hand, and an objectification of bodily movements on the other. These patients are unable to realize their bodily intentions in a synchronized way. The bodily actions are not constituted as a whole, but rather pieced together as successions of singular, fragmented movements. They are not proceeding automatically but become slow, angular and rigid; they also inhibit the patients’ intentional efforts. The link between basic self-awareness and the tacit intentionality of movement is disrupted. As a result of this disconnection, the patients are not able to initiate, plan and structure their directed bodily actions coherently anymore.

In Sass’ phenomenological view, the hyperreflexivity described by the patients contributes much to the fact that their self-experience is becoming opaque and reified [12]. The normally automatic intentional acts are not tacit and unquestioned anymore; they become apparent, hypersignificant and spatialized. Phenomenologically, it is a central property of the second-level self-consciousness to enable the thematization and thus objectification of experiences such as the act of perception or an affective quality thereof. In the patients’ cases, this objectifying dimension of self-consciousness seems to be radicalized in such a way that the previously unthematic components of action are not only rendered thematic, but strictly speaking reified. They are turned into fragmented entities that need to be dealt with individually in order to create meaningful sequences of action or perception.

#### C: Dissociation of operative and active-explicit intentionality

The next stage is reached when the gap between the two layers of intentionality amounts to a disconnection of the first-level pre-reflective bodily self-awareness and the second-level reflective self-consciousness. The patients lose the tacit form of acting and their bodies do not serve as a medium for interacting within the world anymore. In parallel to this development, the pre-reflective SA linked to the implicit experience of bodily actions separates from the higher-order SA linked to the conscious attribution of movement. In fact, patients experience the beginning of a change in the structure of their intentional bodily acts. This progression ends in a state where all automatic performances become explicitand disintegrated. According to Stanghellini [57], patients at this stage do not experience intentional acts such as movements as their own, i.e. through their own sensory awareness, but rather through an alienated, disembodied or *noetic* consciousness [57, p. 128]. They often describe themselves as deanimated bodies ('cyborgs') or disembodied spirits ('scanners'). Schizophrenia patients who in this way feel like a cyborg said:

“If the mind is empty it works like a plotter or a photocamera.” [57, p. 154]

“The computer has been deleted and my brain cells have died. I don’t feel any emotions, because the emotions are located in the right hemisphere of my brain and the right computer has been deleted.”

In both cases, the tacit, automatically synthesized unity of body and mind is interrupted, leading to a feeling of inanimateness [57]. Consequently, the patient experiences his bodily perceptions and movements as alienated:

1. “*For me it was as if my eyes were cameras, and my brain would still be in my body, but somehow as if my head were enormous, the size of a universe, and I was in the far back and the cameras were at the very front. So extremely far away from the cameras. And I walk, and I look around* … *and I’m dizzy and all is like a machine. … I just didn’t have much control over myself*. …, *I have this robot-like feeling in my head, to be looking through cameras, and you observe your whole body, and the steps you take towards the bench*.” [75, pp. 329-330]
2. “The body is something that functions, not something that’s mine, that *I* *live* .” [37, p. 150]

From a phenomenological point of view, both patients suffer from a disembodiment [6,57,74] of the active-explicit intentionality of bodily movement. A gap between the subjects’ selves and their directed bodily activities occurs. Patients then often experience themselves as robots or human machines, or they become passive spectators of their body, thus showing a 'Cartesian' separation of the body and mind. The concept of the reification of the human body and mind was first introduced and described by Descartes in the early 17^th^ century. He conceived of the human body as a mechanical apparatus and explained its functions using physical, chemical and materialist vocabulary [58,85,86]. In line with this concept, the above mentioned two cases also illustrate what may be called “desautomatization of motor action” [9, p. 127]. At this stage, directed bodily movements lose their automaticity and transparency. Moreover, the patients suffer a loss of the sense of bodily agency. Such alienated experiences of one’s own body are a presupposition for the loss of ego-boundaries and the externalisation of the agency (see below). Thus, Conrad described a patient who was trapped into a self-referential and solipsistic experience, for which he coined the term “*Beeinflussungsstimmung*” (mood of influence) [3]. According to Koehler’s “passivity continuum”, this state is characterized by a feeling that “something is going on” within the patient [78, p. 239. Moreover, she has the impression “as if” someone was trying to invade her inner world and to destroy her self-integrity. However, she is not yet convinced that this might actually be true.

#### D: Alienation and externalisation of agency

At the last stage, the intentional arc of bodily movement dissolves; patients’ lose their sense of agency which is instead ascribed to some external force. Impulses and motor actions are no longer experienced as self-generated, but as made impulses and experiences. A patient suffering from schizophrenia coined the term “existential permeability” and described this novel experiential state as follows: “Actions, emotions, and thoughts become strange to the individual, so he attributes them to an external agency, a third-party who controls everything he experiences. (…) In fact, all 3 types of disturbances in existential permeability are rooted in a silenced and diminished sense of self-consciousness, a disappearing awareness that is not part and parcel of ones ego anymore.” [87, p. 1035]. In Stanghellini’s phenomenological view, through such a fundamental alteration, schizophrenia subjects experience a bizarre depersonalisation: their own body becomes “a thing-like mechanism”, inside of which mental life takes place, but as if it came from somewhere in external space [57]. Their intentional impulses and bodily movements are now reversed and directed towards them from an external agent. At this stage, a definite transition into psychosis and FRS occurs.

The following quotation of a schizophrenia patient exemplifies this:

(1) “When I ate this morning, I felt as if somebody else’s head would also be there and would eat with me. It feels like other people would stick their head into my head. When I am chewing, it seems that another tongue comes and takes the food.” [88, p. 1036,^89^]

Another patient with schizophrenia who feels like a “mechanical man” [88, p. 1036] described his experience as follows:

(2) “When I do some work, like swabbing, somebody else is working with me. Just like another person stepped in … It is as if they would be able to put their head in yours. When you move they grab; sometimes they are scared that you would fall. They try to help you, but they are clumsy. (…) It is hard to control the tongue; they just drop some words on your tongue. They fight like devils. My tongue is heavy, like if somebody would hold it.” [88, p. 1036]

Another patient suffering from full-blown schizophrenia said:

(3) “My fingers pick up the pen, but I don’t control them. What they do has nothing to do with me (…) The force moved my lips. I began to speak. The words were made for me” [90, p. 408]

All three patients experience alienation of their own bodily movements. They suffer from a profound change in the phenomenological structure of their experience. It feels like being far away from the lived body. Consequently, they also suffer from a disconnection of normally unified intentional acts such as moving one’s facial muscles in order to eat and the kinaesthetic feedback of these muscles. The synthesis of the initiative to eat, to work or talk to somebody and the corresponding bodily action (moving the jaw, arms or tongue) is not achieved. The intentional arc which normally connects both components is torn apart, and they seem to exist independently of each other. As a result, the patients experience their bodily actions as initiated by an external agent. As a result, the form of the self-experience affects the change in content (e.g. bizarre delusions). In the case of made impulses, they experience a sudden drive to carry out some action as coming from the outside. Thus, both bodily drives and movements may appear as externalized and manipulated. A new hidden intentionality takes rule over the patients’ disembodied volitions and impulses. However, this other subjectivity is still hidden, beyond recognition. In other cases with flamboyant psychotic symptoms, patients refer to an identifiable source of "made" acts (case 4-6) and "made" impulses (case 7):

(4) “When I reach my hand for the comb it is my hand and arm which move, and my fingers pick up the pen, but I don’t control them … I sit there watching them move, and they are quite independent, what they do is nothing to do with me … I’m just a puppet who is manipulated by cosmic strings. When the strings are pulled my body moves and I cannot prevent it.” [1, p. 18]
(5) “Airport security staff had implanted a microchip and a microphone into my neck to control and observe my behaviour and actions. They used these devices to listen to my thoughts and to control me because of my previous suicidal attempts.” (own patient case [58, p. 98])
(6) “They inserted a computer in my brain. It makes me turn to left or right.” [90, p. 408]
(7) “A 26 year old engineer emptied the contents of a urine bottle over the ward dinner trolley. He said: The sudden impulse came over me that I must do it. It was not my feeling, it came to me from the X-ray department, that was why I was sent there for implants yesterday. It has nothing to do with me, they wanted it done. So I picked up the bottle and poured it in. It seemed all I could do.” [1, p. 17]

In the first three cases (case 4-6), due to alienation and externalisation of agency, a second, overwhelming intentionality takes over and causes the patients’ bodily actions. Not until this particular stage of the illness, the phenomenological and clinical distinction between made impulses and made volitional acts is possible: According to Gallagher [64], the first three patients experience the SA on both levels as alienated and externalised. The last patient, however, experiences an impulse to empty the urine bottle. He recognizes himself as being the agent of the sudden behavior. On the other side, he feels being driven from outside to do so. Pointing to the distinction between SO and SA, the last patient suffers from alienation and externalisation of SA as higher-order experience that is linked to the attribution of agency. Although made from outside, he is still able to reflect on his action and recognizes it as his own. More precisely, in this case, SO remains complete extant. Although similar in content, case 1-3 and case 4-6 illustrate different subsets of the psychotic process leading to full-blown psychosis and FRS.

Taken together, however, all four patients are unable to actively intend their bodily actions; rather, they are passively moved and pushed to realize motor activities by an external force. Instead of acting, they feel acted upon. The manipulating agent is not hidden anymore, but gains a concrete, though technical form such as a computer, cosmic strings or an X-ray department. In some cases, there is even a personalized agent [11]. At this stage of the illness, patients experience full-blown schizophrenic symptoms, like FRS.

In summary, from a phenomenological point of view, the transition to frank psychosis occurs in the course of alienation and externalisation of both the level of active-explicit intentionality and the sense of agency after the loss of the anchoring centre of ipseity [91]: At the beginning, the patients’ impulses and bodily actions are not embedded in the first person perspective anymore. Parallel with motoric fragmentation and hyperreflexivity, drives to carry out actions and bodily movements become disembodied, alienated and finally externalised. The patients lose control over their body, and its actions now emanate from an external power.

## Discussion and Clinical Implications

This paper aimed at investigating the self-experience of schizophrenia patients with body-affecting Schneiderian FRS. We have also been looking for the fundamental pattern that characterizes the subjectivity of schizophrenia patients with particular FRS. Two main findings emerged: (1) The subjective experience of made volitional acts and made impulses in schizophrenia is based on a fundamental transformation of both levels of intentionality and sense of agency(2). The above mentioned two FRS are, although clinically very different, of a similar phenomenological structure.

Since the past decade, there is growing empirical evidence that disorders of basic self-awareness are a core feature of schizophrenia spectrum disorders [80,92–94]. We showed that non-psychotic anomalous bodily experiences are rooted in the weakness of operative intentionality. However, only by disembodiment, alienation and finally externalisation of agency the final transition to full-blown psychotic symptoms such as FRS or delusions of alien control occurs. Keeping this psychopathological trajectory in mind, we conclude that the above mentioned FRS are based on a transformation of both levels of intentionality and agency; they should therefore be strongly considered as pathognomonic for the diagnosis of schizophrenia.

On the basis of the present study and other papers investigating FRS in schizophrenia spectrum disorders, assessing psychotic symptoms such as FRS by means of phenomenological psychopathology in daily clinical routine might have some relevant implications: Patients’ statements about their mental state are highly polysemic and their interpretation remains a challenge. In general, a phenomenological approach and careful interviewing might help to extract the salient profile of the psychic disorder behind the patients’ statements (e.g. FRS) [95]. Second, in contrast to operationalized approaches, the phenomenological method is person-centered and emphasizes an authentic and patient-friendly examination of anomalous subjective experience, in spite of the stressful situation it may cause. In fact, the phenomenological approach to interviewing might help to improve patient compliance and thereby positively influence the course of the disease. Third, phenomenological psychopathology has the potential to overcome the Cartesian dualism of mind-versus-body and mind-versus-world still prevalent in daily clinical routine. Fourth, the implementation of phenomenological approaches for studying particular psychotic symptoms can contribute to bridging the explanatory gap between clinical symptoms and current neurobiological models as exemplified by recent studies [96,97]. Fifth, phenomenological accounts of psychotic experience might complement the psychotherapeutic treatment of schizophrenia patients by providing novel strategies to strengthen pre-reflective self-awareness [98]. Finally, the sceptical stance of phenomenological psychopathology might balance the danger of modern psychiatry becoming too reductionist and distant from the individual patient’s experience [95].

We acknowledge potential limitations of the phenomenological method in general and our qualitative study of FRS in particular(1). The patients’ quotations in this paper are of different quality(2). It is important to bear in mind that the investigation of patients’ subjective life depends on their ability to introspect, describe and communicate the symptoms which they are suffering from. In addition, patients might be suspicious, indifferent and not willing to speak about their private and often embarrassing experiences(3). The clinical presentation and the statements which the interviewer is confronted with might be interpreted in different ways (e.g. psychopathologically, dynamically, psychologically etc.)(4). A precise phenomenological exploration requires clinical expertise, time, effort and skill to empathize with the interviewee(5). Last but not least, patients suffering from disorders of pre-reflective self-awareness might have difficulties to articulate their experiences [74,99]. Here, a phenomenologically informed vocabulary could also help patients to arrive at a more detailed and accurate description of their condition.

In this paper we showed that Husserl’s phenomenology is useful to provide a descriptive account and theoretical framework for further psychopathological research on FRS. It is a well-known fact that the clinical distinction between acute psychotic experience in schizophrenia and bipolar or manic disorders in which patients might exhibit risky behavior is very difficult. In daily clinical practice, the differentiation between these two disorders might even be impossible. Furthermore, psychiatric exploration alone cannot pinpoint the underlying theoretical basis of these entities. However, the information about the 1^st^-person experience and the developmental trajectory of psychotic symptoms might have far-reaching consequences regarding the treatment. Therefore, because of its clinical impact, further phenomenological studies on intentionality and sense of agency in patients with manic disorders are needed.

We also suggest that our model contributes to the interdisciplinary investigation of schizophrenic disorders. In order to develop an empirical pluralistic model of psychiatric illnesses and to overcome the above-mentioned limitations, future studies should combine the subject-oriented first-person approach with objectifying third-person approaches such as magnetic resonance imaging (MRI) or genetic analysis. For example, MRI studies might be helpful to investigate strictly categorized subgroups of schizophrenia patients with particular FRS and to demonstrate that impairments of intentionality of bodily movement on a particular level also have neurobiological correlates. Interestingly, recent neuroimaging studies were able to link both specific psychopathological symptoms [100–105] as well as the deficit subtype of schizophrenia [106,107] to alterations in brain structure and function. Among the variety of MRI analysis techniques available at present, resting-state functional MRI (fMRI), an approach that aims to identify temporally synchronous networks or “modes” characterized by ongoing spontaneous modulations of the blood oxygen level during resting-state conditions, seems to be particularly suitable for the investigation of psychopathological symptoms such as FRS since it does not – in contrast to task-based fMRI – require any explicit experimental input or stimulus variation, thus providing a way to remove potential confounds of task performance in patient samples [108].

In conclusion, we propose that the subjective experience of schizophrenia patients with body-affecting FRS is rooted in the disturbance of intentionality and diminished sense of agency. Furthermore, both FRS are, although clinically very different, of a similar phenomenological structure. Although our phenomenological model does not provide a final explanation of FRS, it can stimulate questions that might initiate further theoretical and empirical research in this field.
